# Improved early detection of ovarian cancer using longitudinal multimarker models

**DOI:** 10.1038/s41416-019-0718-9

**Published:** 2020-01-15

**Authors:** Harry J. Whitwell, Jenny Worthington, Oleg Blyuss, Aleksandra Gentry-Maharaj, Andy Ryan, Richard Gunu, Jatinderpal Kalsi, Usha Menon, Ian Jacobs, Alexey Zaikin, John F. Timms

**Affiliations:** 1 Department of Women’s Cancer, Institute for Women’s Health, University College London, Gower Street, London, WC1E 6BT UK; 2 Department of Paediatrics, Sechenov University, Moscow, 119146 Russia; 3 School of Physics, Astronomy and Mathematics, University of Hertfordshire, College Lane, Hatfield, UK; 4 MRC Clinical Trials Unit at UCL, 90 High Holborn, London, WC1V 6LJ UK; 5 President and Vice-Chancellor’s Office, University of New South Wales, Sydney, NSW 2052 Australia; 6 Department of Mathematics, University College London, London, W1T 7DN UK; 7 Department of Applied Mathematics, Lobachevsky State University of Nizhniy Novgorod, Nizhniy Novgorod, 603022 Russia

**Keywords:** Diagnostic markers, Cancer screening, Ovarian cancer, Proteomic analysis

## Abstract

**Background:**

Ovarian cancer has a poor survival rate due to late diagnosis and improved methods are needed for its early detection. Our primary objective was to identify and incorporate additional biomarkers into longitudinal models to improve on the performance of CA125 as a first-line screening test for ovarian cancer.

**Methods:**

This case–control study nested within UKCTOCS used 490 serial serum samples from 49 women later diagnosed with ovarian cancer and 31 control women who were cancer-free. Proteomics-based biomarker discovery was carried out using pooled samples and selected candidates, including those from the literature, assayed in all serial samples. Multimarker longitudinal models were derived and tested against CA125 for early detection of ovarian cancer.

**Results:**

The best performing models, incorporating CA125, HE4, CHI3L1, PEBP4 and/or AGR2, provided 85.7% sensitivity at 95.4% specificity up to 1 year before diagnosis, significantly improving on CA125 alone. For Type II cases (mostly high-grade serous), models achieved 95.5% sensitivity at 95.4% specificity. Predictive values were elevated earlier than CA125, showing the potential of models to improve lead time.

**Conclusions:**

We have identified candidate biomarkers and tested longitudinal multimarker models that significantly improve on CA125 for early detection of ovarian cancer. These models now warrant independent validation.

## Background

Ovarian cancer is the sixth most common cancer in women, causing 152,000 deaths worldwide annually. The overall 5-year survival rate is ~40% due to late presentation, with the majority of cases diagnosed at stage III and IV, where the 5-year survival rate is only 3–19%. Stage I and II ovarian cancers have 5-year survival rates of 40–90%.^1^ Earlier detection of ovarian cancer, in particular aggressive tumours, is a possible way to improve outcomes.

Ovarian malignancies can be divided into two types differing by origin and molecular subtype and are associated with differing prognosis.^2,3^ Type I cancers can arise from tumours of low malignant potential, remain low grade, are slower growing and have a more favourable outcome. Type II cancers are typically high grade, aggressive, associated with poor survival, and are characterised by loss-of-function mutations of TP53 and BRCA1/2 and thus display genomic instability.^4^ It is well established that Type II cancers (mainly high-grade serous cancers) account for most ovarian cancer mortality and early detection of these tumours is likely to translate into mortality benefit.

Two tests used clinically to detect ovarian cancer are serum cancer antigen 125 (CA125) and transvaginal ultrasound. Both have limitations of specificity and sensitivity. CA125 is often elevated in benign conditions such as endometriosis and ovarian cysts and is not always detectable in early-stage disease. This has limited CA125’s potential as an accurate biomarker for early detection.^5–8^ Human epididymis secretory protein E4 (HE4/WFDC2), another Food and Drug Administration-approved biomarker for differential diagnosis of ovarian cancer, has similar limitations in detecting early and asymptomatic cancers.^9–11^

Multimarker tests have been shown to improve performance for ovarian cancer diagnosis compared to CA125 or HE4 alone.^12,13^ For example, the Risk of Ovarian Malignancy Algorithm, combining CA125 and HE4 for determining malignancy during pre-operative assessment, had a sensitivity of 88% compared to 63% and 78%, respectively, for CA125 and HE4 alone.^14–16^ Of more relevance, it has been shown that annual screening using serial serum measurements of CA125 in the Risk of Ovarian Cancer Algorithm (ROCA) within the UK Collaborative Trial of Ovarian Cancer Screening (UKCTOCS) provides an increased detection rate over CA125 cut-off models with a stage-shift in the detected cancers.^17,18^ Although the trial did not report a significant benefit in mortality through screening, there was an indication of a 15% mortality benefit, which awaits confirmation with further follow-up.^19,20^ Given the limitations of current ‘gold standards’ in ovarian cancer detection, there is an urgent need for new biomarkers. The incorporation of these into longitudinal algorithms with CA125 may be a promising strategy for improving early detection of ovarian cancer. We have previously described the Method of Mean Trends (MMT) algorithm, which provided high performance in predicting ovarian cancer based on serial CA125 measurements^21^ and have now adapted this to include multiple serial biomarker measurements.

Herein, we present the discovery of potential new serum biomarkers and longitudinal multimarker models capable of discriminating cancer-free controls and ovarian cancer cases prior to clinical diagnosis. We used 490 serial serum samples from 49 ovarian cancer cases taken at different times prior to diagnosis and 31 matched non-cancer controls nested within UKCTOCS, applying multidimensional liquid chromatography tandem mass spectrometry (LC-MS/MS) with tandem mass tagging for biomarker discovery. Promising candidates from this and the literature were assayed in all serial samples from these women and combined with CA125, and the data was used to generate longitudinal multimarker models. Using this novel approach, we show both increased sensitivity for ovarian cancer detection and earlier detection than using CA125 alone.

## Methods

### Sample set

The study set comprised serum from women recruited to UKCTOCS collected according to a standard operating procedure.^17^ Trial participants at enrolment were post-menopausal women aged 50–74 years who had no family history of ovarian cancer. Women subsequently diagnosed with ovarian cancer were identified by cross-referencing with the Health and Social Care Information Centre cancer registry and death codes, with diagnosis confirmed by review of histopathology reports. Forty-nine cases from the multimodal arm of UKCTOCS were selected comprising 10 borderline (BL) cases, 9 Type I cases and 30 Type II cases (Table 1). Thirty-one matched controls from the multimodal arm were selected who had no history of cancer and were matched to Type II cases based on age (±5 years), collection date (within 6 months), and collection centre. All serial samples from these women (*n* = 490) were retrieved from cryostorage, shipped on dry ice to the laboratory, and stored at −80 °C prior to analysis.

**Table 1:** Sample set characteristics.

(A) Characteristics of sample/patient cohort with cancer morphology, grade and Type							
	Cases				Controls		
No. of individuals	49				31		
No. of samples	315				175		
Mean age at sample draw (years) (range)	66.4 (50.6–79.7)				65.0 (51.4–79.2)		
Median time to diagnosis (months) (range)	26.27 (2.27–97.51)				na		
Morphology—grade (Type)							
Serous—High grade (Type II)	23						
Endometrioid—High grade (Type II)	3						
Carcinosarcoma—High grade (Type II)	1						
Not specified—High grade (Type II)	3						
Endometrioid—Low grade (Type I)	5						
Clear cell—High grade (Type I)	3						
Not specified—Low grade (Type I)	1						
Borderline (mixed morphologies)	10						

(B) Characteristics of sample groups used for discovery profiling							
Sample set	Sample group	No. of cases	No. of samples	Mean age (years)	Mean time to diagnosis (months)	Mean BMI	Modal FIGO stage
Discovery	Type I/BL ‘late’	9/10	9/10	68.1/70.1	8.8/4.7	28.4/26.04	Ia/Ia
	Type II ‘late’	30	30	67.1	5.5	26.8	IIIc
	Control ‘late’	31	31	67.1	na	25.7	na
	Type I/BL ‘early’	9/10	9/10	64.8/65.2	48.3/62.8	28.4/26.04	Ia/Ia
	Type II ‘early’	30	30	62.5	60.4	26.8	IIIc
	Control ‘early’	31	31	62.7	NA	25.5	na
All annual samples	Type I	9	36	67.6	30.7	27.9	Ia
	Type II	30	144	64.5	37.8	26.4	IIIc
	Controls	31	158	64.7	na	25.4	na

Only annual screening samples were used for biomarker algorithm development

*BL* borderline ovarian cancers

### LC-MS/MS discovery analysis

Samples were pooled into six groups for MS-based discovery, comprising ‘late’ (<14 months to diagnosis) and ‘early’ (>35 months to diagnosis) samples for each cancer case and control: Type I/BL early and late, Type II early and late, control early, and late (Table 1B). A pooling approach was taken, as the analysis of individual samples with extensive fractionation is not feasible in terms of time and cost-effectiveness. Details of the method can be found in Supplementary Materials and Methods. Briefly, pools were sequentially immunodepleted of the top 20 most abundant serum proteins, digested with trypsin, labelled in 6-plex using TMT reagents, and extensively fractionated (100 fractions) by strong anion exchange and high pH reversed-phase LC, prior to LC-MS/MS analysis on orbitrap instruments, essentially as described.^22^ Raw data files were combined and analysed using the Proteome Discoverer V1.4 software with database searching using the Mascot search engine V2.4. Data were filtered and reporter ion-based relative quantification of protein groups applied to compare expression across the six groups. A biomarker scoring system was applied to aid in candidate selection, ranking the proteins based on magnitude and consistency of expression differences, data quality, and biological function (see Supplementary Materials). The full data set and scoring system is available as Supplementary Data File S1.

### Serum assays

Serum concentrations of biomarker candidates were quantified using commercial enzyme-linked immunosorbent assays (ELISA) or chemiluminescence immunoassays. Kits were first tested on pooled samples according to the manufacturers’ instructions to define optimal dilutions and assay reproducibility. The kits used, catalogue numbers, dilutions, and intra-assay coefficient of variations were: Human AGR2 ELISA Kit (ElabScience; E-EL-H0298; 1:20; 18%), CA125 ECLIA assay (Roche; Elecsys CA 125 II; 1:1; 4%), CHI3L1 Quantikine ELISA Kit (R&D Systems; DC3L10; 1:50; 14%), DNAH17 (human) ELISA Kit (EIAab; E5886h, 1:5; 17%), FSTL1 ELISA Kit (USCN; SEJ085Hu; 1:100; 11%), Glycodelin/PP14 Elisa Kit (Bioserv Diagnostics; BS-30-20; 1:2; 22%), HE4 ECLIA assay (Roche; Elecsys HE 4; 1:1; 8%), LRG1 ELISA Kit (IBL; 27769; 1:2000; 16%), Human PEBP4 ELISA Kit (ElabScience; E-EL-H5440; 1:200; 20%), and SLPI Quantikine ELISA Kit (R&D Systems; DP100, 1:50; 12%).

### Model building, testing and statistical analysis

**Fig. 1:**
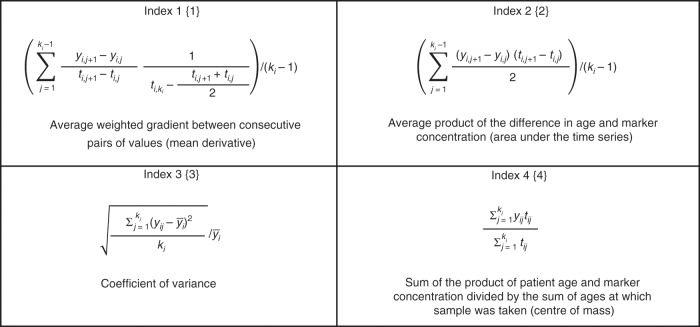
Longitudinal trend indices used for transforming serial data. For every *i*th patient, *k*_*i*_ is the total number of serial measurements, *y*_*i,j*_ is the *j*th serial measurement and *t*_*i,j*_ and *t*_*i,ki*_ represent, respectively, ages at which the current and the most recent measurements were taken. A simple cut-off for the final measurement of each candidate marker was used as Index 5{5}.

R software was used for model building and statistical analysis. *T* test or Mann–Whitney test was performed for parametric or non-parametric values, respectively, as determined by the D’Agostino and Pearson omnibus normality test. All marker values were log10-transformed for model generation. To generate longitudinal, multimarker models, for each individual, serial biomarker values from annual samples (taken <5 years to diagnosis) were first transformed into a single value that represented the degree of change over time of the candidate marker using one of the four indices (Fig. 1). Index 1{1} determines the average weighted gradient between consecutive pairs of values (mean derivative). Index 2{2} is the average product of the difference in age and marker concentration, representing the area under the time series. Index 3{3} is the coefficient of variance and does not use time as a factor. Index 4{4} is the sum of the product of patient age and marker concentration divided by the sum of ages at which the sample was taken (i.e., the centre of mass) and thus would reduce any effect of age on marker concentration, should such a relationship exist. We have previously described these indices and used them to build the MMT algorithm, based on serial CA125 measurements.^21^ Here indices were applied to all candidate measurements and, together with raw measurements (Index 5{5}), subjected to variable selection using a robust methodology that included Akaike Information Criterion (AIC), least absolute shrinkage and selection operator (lasso), bootstrapped lasso, mean accuracy decrease, and Gini impurity. AIC selection was performed using the standard implementation from package MASS in R. For lasso, the penalty parameter lambda was chosen as the first value which left only 3 variables in the model starting from 0 with 0.01 steps. In bootstrap lasso, 200 re-samplings were created from the original data and the traditional lasso approach applied to each of them. For each variable, the frequency of inclusion in the top three re-samplings was reported. Standard implementation of the mean accuracy decreases and Gini impurity was used from the randomForest package in R. This process identified the variables that could potentially serve as ‘good’ predictors in our models. All combinations of these predictors were then tested as logistic regression models, limiting the number of variables to three to avoid overfitting. The Caret package in R was used for leave-one-out cross-validation for selection of the best models. Models were then fitted on the whole data set and comparisons of sensitivity for the longitudinal models at fixed high specificity (>90% and >95%) were performed using McNemar’s test. Confidence intervals for sensitivity were generated by 2000 stratified bootstrap replicates^23^ for visual comparison. Model calibration was assessed with the Hosmer–Lemeshow goodness-of-fit test, with *P* values < 0.05 indicating a poorly calibrated model.

## Results

### MS-based profiling of pre-diagnosis serum samples

A set of 490 serum samples was sourced from the UKCTOCS biobank comprising serial samples from 49 volunteers who were later diagnosed with BL (*n* = 10), Type I (*n* = 9) or Type II (*n* = 30) ovarian malignancy (Table 1). Serial samples were also taken from matched cancer-free controls (*n* = 31). All women were aged >50 years and post-menopausal with no significant difference in age between the case and control groups. The majority of Type II cancers were high-grade serous (*n* = 23) with Type I cancers comprising low-grade endometrioid (*n* = 5), clear cell (*n* = 3) and low-grade unspecified (*n* = 1). Paired samples from these volunteers (*n* = 160) taken <14 months (‘late’) and >35 months (‘early’) prior to diagnosis and from matched controls (Table 1B) were pooled by cancer Type and time group and subjected to immunodepletion, tryptic digestion, 6-plex TMT-labelling and extensive peptide fractionation prior to LC-MS/MS-based proteomic profiling. TMT labelling efficiency was >99% with labelling in each TMT channel between 91.3% and 92.8%. This analysis yielded 748 protein groups quantified across all six sample groups. There were notable differences in protein expression and scores between the Type I/BL and Type II groups, possibly reflecting differences in tumour molecular profiles and the heterogeneity of the Type I/BL cases. Particularly, several acute-phase response proteins (e.g., A1AT, CRP, HP, ORM1, ORM2 and SAA1) were more highly upregulated in the Type I/BL group towards diagnosis, suggesting that Type I and/or BL disease is characterised by a more inflammatory phenotype.

### Candidate selection and univariate testing

**Fig. 2:**
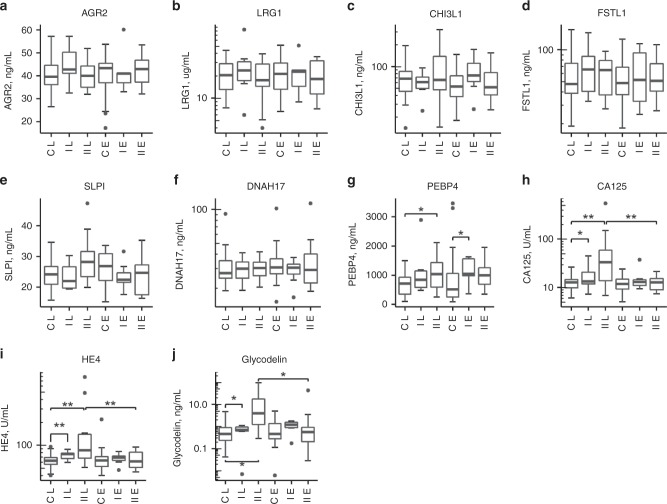
Tukey box and whisker plots showing changes in pre-diagnostic serum candidate biomarker levels in cases and controls in two-time groups; <14 months to diagnosis (late) and >35 months to diagnosis (early). **a** AGR2 serum levels; **b** LRG1 serum levels; **c** CHI3L1 serum levels; **d** FSTL1 serum levels; **e** SLPI serum levels; **f** DNAH17 serum levels; **g** PEBP4 serum levels; **h** CA125 serum levels; **i** HE4 serum levels; **j** glycodelin serum levels. *P < 0.05, **P < 0.001 from either *t* test or Mann–Whitney test. Data for borderline cases are omitted. Axis labels: C = controls; I = Type I; II = Type II; L = late; E = early.

Five high-scoring candidates were selected for further testing based on functional assignment and availability of suitable commercial assays: chitinase-3-like protein 1 (CHI3L1/YKL40), dynein heavy chain 17 (DNAH17), follistatin-related protein 1 (FSTL1), leucine-rich alpha-2-glycoprotein (LRG1), and phosphatidylethanolamine-binding protein 4 (PEBP4). Our previous identification of CHI3L1 as a possible marker of early ovarian epithelial cell transformation^24^ also supported the choice of candidate. Four further proteins were selected based on previous studies: anterior gradient protein 2 (AGR2) homologue,^25^ human epididymis 4 (HE4/WFDC2),^14,26^ glycodelin (PAEP),^27–29^ and antileukoproteinase (SLPI).^30,31^ Assays were performed on all 490 serial samples from the 80 cases and controls to generate serial data (Supplementary Material; Fig. S1). Measurements for serum CA125 taken from UKCTOCS were also included in the analysis. We found no correlation between the concentration of any candidate and time from sample collection to spin (Pearson’s correlation coefficient, *R* < |0.2|), discounting any possible confounding effects of sample processing. In comparing late and early time groups used for LC-MS/MS discovery, CA125, HE4 and glycodelin distinguished cases from controls in the late time group and between the late and early time groups for Type II cases (Fig. 2h–j). PEBP4 distinguished Type II cases from controls in the late group (*P* = 0.02) and Type I cases from controls in the early group (*P* = 0.034) (Fig. 2g). No other candidate gave significant differences (*P* < 0.05), although trends were evident for some.

### Generation and testing of multimarker longitudinal models

Longitudinal analysis may increase the predictive capability of biomarkers.^18^ To address this, we first binned samples into pre-diagnosis time groups of ≤15, 15–30, 30–45 and >45 months and tested logistic regression models using log-transformed values, including up to 3 marker candidates or epidemiological variables (age, body mass index, oral contraceptive pill use, and hormone replacement therapy use) in the models. After leave-one-out cross-validation, these models failed to show any clinically significant improvement on CA125 alone (Supplementary Material; Table S1). We therefore undertook a novel longitudinal approach,^21^ whereby four trend indices ({1}–{4}) describing changes in concentration over time were used to transform the measurements for each candidate into a single value (Fig. 1). Only data from annual screening samples was used to eliminate potential bias from repeat CA125 testing triggered by ROCA in UKCTOCS and data for BL cases were also excluded. Summary statistics for the indexed biomarker values are presented in Tables S2 and S3.

Multimarker models may improve classification over single-marker tests. Therefore, following rigorous variable selection (Table S4), logistic regression was used to combine longitudinal index-transformed marker values, using a maximum of three main effects to avoid overfitting and applying leave-one-out cross-validation. Models were then evaluated using all cases whose final annual screening sample fell within 1 year to diagnosis. Samples taken >5 years before diagnosis were also excluded, leaving serial samples from 6 Type I and 22 Type II cases for comparison with serial samples from the 31 controls. Median sensitivities at fixed specificity (90.3% and 95.4%) were determined for each model with 95% confidence intervals (CIs) calculated by bootstrapping. Goodness-of-fit testing showed that the CA125{5} model was poorly calibrated (*P* = 0.04), while all multimarker models were well calibrated (*P* values ranging from 0.343 to 0.975). Model sensitivities were then compared to CA125 cut-off model CA125{5}, using McNemar’s exact test.

**Fig. 3:**
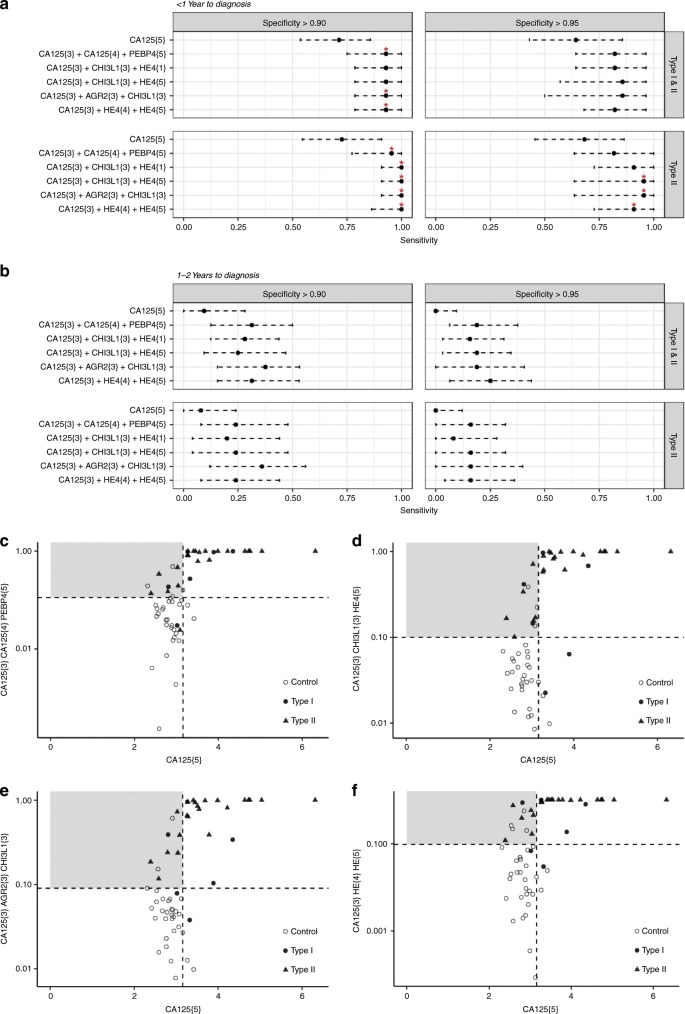
Top five longitudinal models. **a** Sensitivity of models with 95% CI at 90.3% (left) and 95.4% (right) specificity at <1 year to diagnosis for all cases (top) or Type II cases only (bottom). Sensitivities were compared to CA125 cut-off model (CA125{5}) by one-tailed McNemar’s test; *P < 0.05. **b** Sensitivity of models 1–2 years before diagnosis. **c**–**f** Predictions are presented as logistic regression outcomes for four models plotted against model CA125{5}. Horizontal and vertical dashed lines represent cut-offs for classification at 90.3% sensitivity. Cases in the grey box were identified by the multimarker model and not by CA125{5}.

Considering all cases, three models combining CA125 with PEBP4, AGR2, CHI3L1 and/or HE4 gave significantly higher sensitivities (*P* < 0.05) at 90.3% specificity up to 1 year before diagnosis than CA125 alone (Table 2A, Fig. 3a, Table S5). At 95.4% specificity, while no model was significantly better than CA125 (best *P* value = 0.055), all had sensitivities >82%; the 2 best models (CA125{3}AGR2{3}CHI3L1{3} and CA125{3}CHI3L1{3}HE4{5}) had 87.5% sensitivity at 95.4% specificity, missing only 2 cases (Fig. 3d, e). Considering Type II cases only, 4 models gave 100% sensitivity at 90.3% specificity (Table 2 and Fig. 3a) and all models performed significantly better than CA125 alone (Table S5). The two best models (above) provided 95.5% sensitivity at 95.4% specificity (Table 2B and Fig. 3a), significantly outperforming CA125 (Table S5). Thus we demonstrate longitudinal multimarker models with significantly improved performance over CA125 alone for detecting ovarian cancer up to 1 year before diagnosis.

**Table 2:** Performance of top models based on leave-one-out-cross-validation.

Model	AUC (95% CI)	Sensitivity (95% CI) at 0.903 specificity	Sensitivity (95% CI) at 0.954 specificity
(A) All cases up to 1 year to diagnosis			
CA125{5}	0.869 (0.775–0.962)	0.714 (0.536–0.857)	0.643 (0.429–0.857)
CA125{3}CA125{4}PEBP4{5}	0.96 (0.923–0.997)	0.929 (0.75–1)	0.821 (0.643–0.964)
CA125{3}CHI3L1{3}HE4{1}	0.959 (0.916–1)	0.929 (0.786–1)	0.821 (0.643–0.964)
CA125{3}CHI3L1{3}HE4{5}	0.96 (0.922–0.998)	0.929 (0.786–1)	0.857 (0.571–0.964)
CA125{3}AGR2{3}CHI3L1{3}	0.966 (0.938–0.995)	0.929 (0.786–1)	0.857 (0.5–0.964)
CA125{3}HE4{4}HE4{5}	0.974 (0.95–0.997)	0.929 (0.786–1)	0.821 (0.679–0.964)
(B) Type II cases up to 1 year to diagnosis			
CA125{5}	0.868 (0.756–0.979)	0.727 (0.546–0.909)	0.682 (0.455–0.864)
CA125{3}CA125{4}PEBP4{5}	0.97 (0.934–1)	0.955 (0.773–1)	0.818 (0.636–1)
CA125{3}CHI3L1{3}HE4{1}	0.986 (0.973–0.999)	1.0 (0.909–1)	0.909 (0.727-1)
CA125{3}CHI3L1{3}HE4{5}	0.984 (0.97–0.998)	1.0 (0.909–1)	0.955 (0.636–1)
CA125{3}AGR2{3}CHI3L1{3}	0.984 (0.971–0.998)	1.0 (0.909–1)	0.955 (0.636–1)
CA125{3}HE4{4}HE4{5}	0.988 (0.976–1)	1.0 (0.864–1)	0.909 (0.727–1)
(C) All cases at 1–2 years to diagnosis			
CA125{5}	0.507 (0.382–0.631)	0.094 (0–0.281)	0 (0–0.094)
CA125{3}CA125{4}PEBP4{5}	0.658 (0.543–0.772)	0.313 (0.125–0.5)	0.188 (0.063–0.375)
CA125{3}CHI3L1{3}HE4{1}	0.648 (0.531–0.765)	0.281 (0.125–0.438)	0.156 (0.031–0.313)
CA125{3}CHI3L1{3}HE4{5}	0.65 (0.54–0.789)	0.25 (0.094–0.469)	0.188 (0.031–0.344)
CA125{3}AGR2{3}CHI3L1{3}	0.697 (0.595–0.8)	0.375 (0.156–0.532)	0.188 (0–0.406)
CA125{3}HE4{4}HE4{5}	0.649 (0.532–0.766)	0.313 (0.156–0.531)	0.25 (0.063–0.438)
(D) Type II cases at 1–2 years to diagnosis			
CA125{5}	0.504 (0.361–0.648)	0.08 (0–0.24)	0 (0–0.12)
CA125{3}CA125{4}PEBP4{5}	0.632 (0.499–0.765)	0.24 (0.08–0.48)	0.16 (0.04–0.32)
CA125{3}CHI3L1{3}HE4{1}	0.65 (0.523–0.778)	0.2 (0.04–0.44)	0.08 (0–0.28)
CA125{3}CHI3L1{3}HE4{5}	0.643 (0.519–0.766)	0.24 (0.04–0.48)	0.16 (0–0.32)
CA125{3}AGR2{3}CHI3L1{3}	0.71 (0.6–0.82)	0.36 (0.12–0.56)	0.16 (0–0.4)
CA125{3}HE4{4}HE4{5}	0.644 (0.517–0.772)	0.24 (0.08–0.44)	0.16 (0.04–0.36)

Area under the ROC curve (AUC) is given with 95% confidence intervals (CI) calculated using the method of DeLong. Sensitivities are given at specificities of 0.903 and 0.954 with 95% CI calculated by bootstrapping with 2000 stratified replicates

**Fig. 4:**
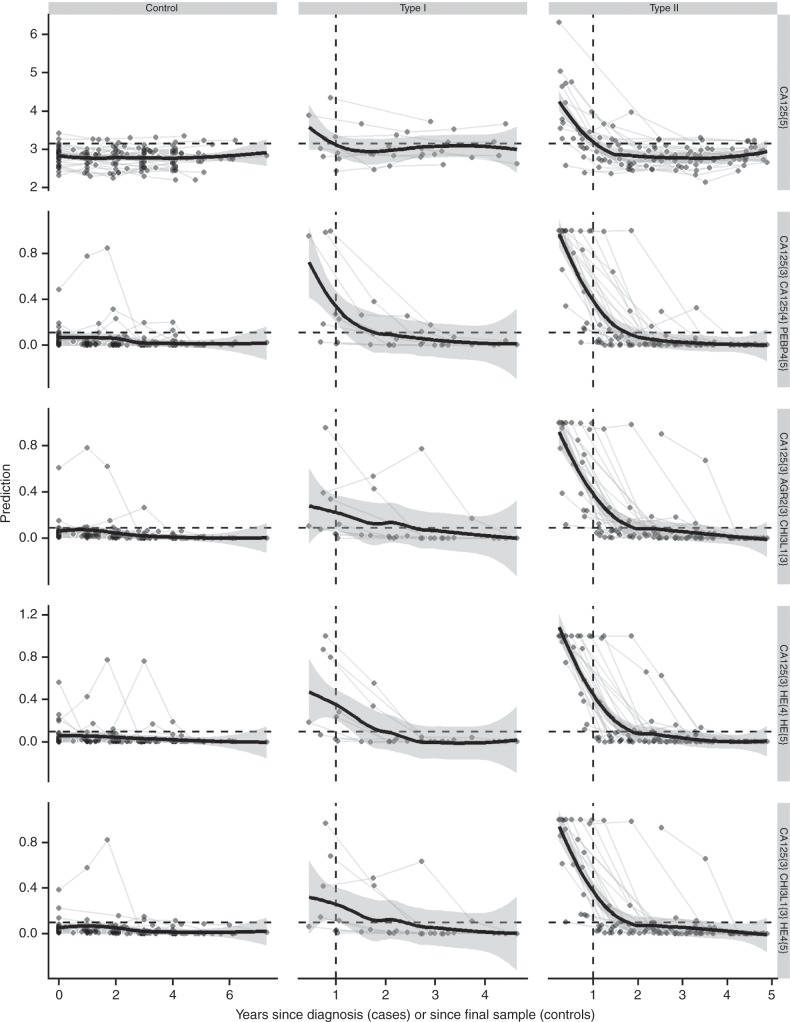
Model predictions presented as logistic regression outcomes for multivariate and raw outcome for univariate analyses by time. Time is years to diagnosis for cases and years since final sample for controls. Model cut-offs giving 90.3% specificity are indicated by horizontal dashed lines. Grey lines represent individuals and circles the time the sample was taken. The thick line is a Loess fit over all points with 95% CIs in grey shading. Vertical dashed lines indicate the timepoint at which the Loess curve intersects the threshold for model CA125{5}.

The capacity of models to predict ovarian cancer earlier than 1 year before diagnosis was next investigated. Performances were evaluated within 1–2 years of diagnosis after excluding samples taken within 1 year of diagnosis. All longitudinal models had sensitivities >25% at 90.3% specificity, although none were significantly better than CA125 alone (Table 2C, Fig. 3b, Table S5). The best model (CA125{3}AGR2{3}CHI3L1{3}) provided 37.5% sensitivity (all cases) and 36% sensitivity (Type II cases) at 90.3% specificity versus 9.4% and 8.0%, respectively, for CA125 alone. Plotting prediction values against time to diagnosis showed that the models detected cancer earlier than CA125 (Fig. 4). Models CA125{3}AGR2{3}CHI3L1{3} and CA125{3}HE4{4}HE4{5} were the best for Type II cases, providing test positivity 18 and 15 months earlier, respectively, than CA125. The data show the potential of the models to improve on the lead time of detection.

## Discussion

While elevated serum CA125 is a reasonably robust marker for detecting ovarian cancer, it is not always raised in early-stage disease and can be elevated in benign conditions. This necessitates the discovery of complementary biomarkers and algorithms that together with CA125 will improve early detection of ovarian cancer. Herein we have identified new candidate biomarkers using a set of pre-diagnosis serum samples nested within UKCTOCS and generated longitudinal data for these markers. The data were then used to develop novel longitudinal models capable of identifying ovarian cancer cases that were undetectable using CA125 alone and gave a significant improvement in performance up to 1 year prior to diagnosis and with improved lead times of detection.

Initial proteomic profiling of pooled sera identified a number of potential biomarkers that were elevated in cancer cases compared to controls. One of these was PEBP4, a suppressor of proliferation and invasion in ovarian tumour cell lines,^32^ and a RAF1- and MEK1-binding protein that inhibits tumour necrosis factor-α-induced apoptosis.^33^ PEBP4 was significantly elevated in sera from cancer cases prior to diagnosis and featured in one of the top multivariable longitudinal models. CHI3L1 also featured in two of the best models, and while we showed it not to be discriminatory alone, it complemented CA125 when used longitudinally. This highlights the importance of not discounting candidates based on univariate analysis alone. CHI3L1 is a carbohydrate-binding lectin with a purported role in tissue remodelling, angiogenesis and survival that may function by modulating chemokine and inflammatory responses. It has been previously reported as a serum marker of ovarian cancer^34^ and, in one study, outperformed CA125 for detecting early-stage disease.^35^ Our previous work had identified elevated levels of secreted CHI3L1 in immortalised ovarian surface epithelial cells transformed with either MYC or MYC plus activated KRAS-G12V, suggesting that its overexpression may be an early event in epithelial cell transformation.^24^ AGR2 also complemented CA125 and CHI3L1 in one of the top models. AGR2 is a secreted and endoplasmic reticulum-resident chaperone protein required for folding, trafficking and assembly of cysteine-rich transmembrane receptors and mucins. Its overexpression in numerous cancer types has been linked with enhanced cell migration and proliferation, altered adhesion and differentiation and promotion of angiogenesis and metastasis.^36^ Previous studies have found elevated tissue expression of AGR2 to be an indicator of poor prognosis,^37^ and it has been reported as a putative blood-borne biomarker for the detection and/or prognosis of ovarian, pancreatic, prostate and lung cancer.^25,38–40^ Our findings support AGR2 as a biomarker for early ovarian cancer detection that complements CA125 (and CHI3L1) when used longitudinally.

A major challenge in early detection is how best to normalise a biomarker to natural variations in serum levels within individuals and across populations. By analysing longitudinal data using the described indices, we have defined changes in biomarker levels that deviate from individual baselines providing greatly improved performance compared to simple cut-off models. This type of approach was used successfully to develop the ROCA, where serial CA125 measurements were used to substantially improve on using single CA125 measurements.^18^ In agreement, the best single marker in our data set was longitudinal CA125 using Index 3 (CA125{3}), which provided higher sensitivity at fixed specificity compared to using the final CA125 measurement, although the increase was not significant. However, by combining longitudinally indexed markers (including CA125), we show significant improvement in sensitivity and lead time over using CA125 alone.

The sensitivity of the CA125 cut-off model used herein (71.4%, 95% CI 53.6–85.7) was similar to that reported previously using a validation set of 25,042 UKCTOCS women (73.1%, 95% CI 63.6–80.8) at 90% specificity.^21^ This demonstrates that the distribution of CA125 in our data set is representative of the whole of UKCTOCS. Comparison with performance of published serial CA125 algorithms applied to the whole UKCTOCS cohort as first-line screening tests shows our models gave higher sensitivity at similar specificity. Four of the models gave 92.9% sensitivity (95% CI 78.6–100) at 90.3% specificity, compared to MMT with 86.5% sensitivity (95% CI 78.4–91.9) at 89.5% specificity, PEB with 88.5% sensitivity (95% CI 80.6–93.4) at 89.5% specificity, and ROCA with 87.1% sensitivity at 87.6% specificity.^18,21^ The best model (CA125{3}AGR2{3}CHI3L1{3}) also substantially outperformed these serial CA125 algorithms at 1–2 years to diagnosis, with a sensitivity of 37.5% versus 23.3% for MMT and 26.7% for PEB,^21^ detecting cancers earlier. Moreover, for the more aggressive Type II cases, our models provided 100% sensitivity at 90.3% specificity at 1 year to diagnosis. Thus we have demonstrated that combining additional markers longitudinally improves detection rate and lead time compared to using CA125 alone.

The key strengths of our study are the use of a novel combination of longitudinal biomarker measurements using pre-diagnosis serial samples from a well-characterised cohort, with rigorous variable selection and model testing that compared poor prognosis cancers with all cancer cases. The main weakness of this discovery study was the small sample size used, with samples from 6 Type I and 22 Type II cases used for model testing; some cases were excluded as they lacked an annual UKCTOCS sample taken within 1 year to diagnosis, and we used controls only from women who had not been diagnosed with any cancer at sample donation and during follow-up; the latter may exaggerate specificity. The small sample size and heterogeneous nature of the cases also precluded any meaningful subset analysis. Despite rigorous variable selection and cross-validation, we cannot rule out the potential for overfitting and it is now essential to validate these models in a larger independent cohort of samples with more detailed investigation of lead time and model performance by stage and histological subtype. Nevertheless, the findings are encouraging and advocate the benefits of incorporating serial sampling into biomarker discovery and clinical testing studies.

In conclusion, we have generated multimarker longitudinal models for the early detection of ovarian cancer that significantly outperform CA125, detecting Type I and II cases that CA125 did not. Furthermore, we show the potential of these models to improve lead time. Blinded validation of these models in a larger, longitudinal sample set is now warranted to investigate the potential of these algorithms for early detection in ovarian cancer screening.

## Supplementary information

Supplemental Material

Dataset S1

## Data Availability

Raw assay data, excepting CA125, are available upon request.
