# Supplementary material for: Improved early detection of ovarian cancer using longitudinal multimarker models
Source: Br J Cancer. 2020 Jan 15;122(6):847–56. doi: 10.1038/s41416-019-0718-9 (PMC7078315; doi:10.1038/s41416-019-0718-9)
Supplement: Supplementary file 1 — Supplemental Material [file 41416_2019_718_MOESM1_ESM.docx]

Supplementary Material

**Supplementary Data**

**Table S1** Logistic regression model performances by pre-diagnosis time group. The area under the ROC curve (AUC) for the three best-performing cross-validated logistic regression models are shown. Models were derived using data from samples taken <15 month to diagnosis and matched controls and are compared in the other time groups shown and with CA125 alone.

| Pre-diagnosis time group | Model 1: CA125, CHI3L1, FSTL1 (leave-one-out cross-validation AUC) | Model 2: Age, CA125, FSTL1 (leave-one-out cross-validation AUC) | Model 3: Age, CA125, PEBP4 (leave-one-out cross-validation AUC) | CA125 (leave-one-out cross-validation AUC) |
| --- | --- | --- | --- | --- |
| <15 months | 0.79 | 0.789 | 0.781 | 0.78 |
| 15-30 months | 0.51 | 0.551 | 0.533 | 0.519 |
| 30-45 months | 0.402 | 0.386 | 0.302 | 0.431 |
| >45 months | 0.501 | 0.51 | 0.492 | 0.547 |

**Table S2.** Mean control and case values for longitudinally-indexed individual marker candidates and variance for Type I and II cases. Annual samples from Type I (n=9) and Type II (n=30) ovarian cancer cases are considered (excluding Borderlines). *P* values were determined by Mann-Whitney U Test (**P*≤0.05).

|  | Index 1 | | | Index 2 | | | Index 3 | | | Index 4 | | | Variance | | |
| --- | --- | --- | --- | --- | --- | --- | --- | --- | --- | --- | --- | --- | --- | --- | --- |
| Candidate | Control | Case | *P* value | Control^Ϯ^ | Case^Ϯ^ | *P* value | Control | Case | *P* value | Control | Case | *P* value | Control | Case | *P* value |
| AGR2 | 0.02 | 0.06 | 0.58 | -0.78 | -0.13 | 0.24 | 0.06 | 0.07 | 0.55 | 3.57 | 3.56 | 0.72 | 0.08 | 0.09 | 0.38 |
| CA125 | 0.01 | 0.37 | <0.001* | 0.16 | 3.12 | <0.001* | 0.03 | 0.11 | <0.001* | 2.76 | 3.11 | <0.001* | 0.01 | 0.29 | <0.001* |
| CHI3L1 | 0.06 | 0.02 | 0.77 | 0.17 | 0.25 | 0.27 | 0.05 | 0.07 | 0.04* | 4.00 | 4.02 | 0.80 | 0.07 | 0.15 | 0.03* |
| DNAH17 | 0.02 | 0.09 | 0.06 | -0.15 | 0.46 | 0.11 | 0.07 | 0.07 | 0.57 | 3.55 | 3.55 | 0.46 | 0.12 | 0.10 | 0.65 |
| FSTL1 | 0.02 | 0.05 | 0.49 | 0.15 | -0.06 | 0.48 | 0.04 | 0.05 | 0.01* | 3.97 | 3.98 | 0.78 | 0.03 | 0.06 | 0.05* |
| HE4 | 0.00 | 0.22 | <0.001* | 0.07 | 1.92 | <0.001* | 0.01 | 0.05 | <0.001* | 4.18 | 4.32 | 0.03* | 0.01 | 0.14 | <0.001* |
| PEBP4 | 0.05 | 0.14 | 0.48 | -1.25 | -0.39 | 0.33 | 0.09 | 0.08 | 0.89 | 5.91 | 6.10 | 0.11 | 0.40 | 0.36 | 0.62 |
| SLPI | 0.00 | 0.04 | 0.04* | -0.09 | 0.42 | 0.03* | 0.03 | 0.03 | 0.37 | 3.38 | 3.39 | 0.93 | 0.01 | 0.02 | 0.46 |

^Ϯ^ Values x1000

**Table S3** Mean control and case values for longitudinally-indexed individual marker candidates and variance for Type II cases. Annual samples only from Type II ovarian cancer cases (n=30) are considered. *P* values were determined by Mann-Whitney U Test (**P*≤0.05).

|  | Index 1 | | | Index 2 | | | Index 3 | | | Index 4 | | | Variance | | |
| --- | --- | --- | --- | --- | --- | --- | --- | --- | --- | --- | --- | --- | --- | --- | --- |
| Candidate | Control | Case | *P* value | Control^Ϯ^ | Case^Ϯ^ | *P* value | Control | Case | *P* value | Control | Case | *P* value | Control | Case | *P* value |
| AGR2 | 0.02 | 0.08 | 0.43 | -0.78 | 0.00 | 0.19 | 0.06 | 0.07 | 0.66 | 3.57 | 3.55 | 0.51 | 0.08 | 0.08 | 0.50 |
| CA125 | 0.01 | 0.41 | <0.001* | 0.16 | 3.44 | <0.001* | 0.03 | 0.13 | <0.001* | 2.67 | 3.07 | <0.01* | 0.01 | 0.35 | <0.001* |
| CHI3L1 | 0.06 | 0.03 | 0.95 | 0.17 | 0.83 | 0.09 | 0.05 | 0.08 | 0.02* | 4.00 | 3.97 | 0.51 | 0.07 | 0.15 | 0.03* |
| DNAH17 | 0.02 | 0.10 | 0.06 | -0.15 | 0.73 | 0.07 | 0.07 | 0.07 | 0.92 | 3.55 | 3.54 | 0.39 | 0.12 | 0.11 | 0.99 |
| FSTL1 | 0.02 | 0.04 | 0.74 | 0.15 | 0.05 | 0.65 | 0.04 | 0.05 | <0.001* | 3.97 | 3.95 | 0.96 | 0.03 | 0.06 | <0.001* |
| HE4 | 0.00 | 0.23 | <0.001* | 0.07 | 2.01 | <0.001* | 0.01 | 0.06 | <0.001* | 4.18 | 4.31 | 0.14 | 0.01 | 0.17 | <0.001* |
| PEBP4 | 0.05 | 0.13 | 0.5 | -1.25 | -0.08 | 0.21 | 0.09 | 0.08 | 0.72 | 5.91 | 6.02 | 0.28 | 0.40 | 0.33 | 0.81 |
| SLPI | 0.00 | 0.04 | 0.02* | -0.09 | 0.43 | 0.04* | 0.03 | 0.03 | 0.33 | 3.38 | 3.39 | 0.91 | 0.01 | 0.01 | 0.39 |

^Ϯ^ Values x1000

**Table S4.** Variable selection showing indices appearing among the best performing at least once, ranked for Lasso, Bootstrapped Lasso (BS_lasso), Mean Accuracy decrease (MeanAcc), Gini Impurity (Gini) and Akaike Information Criterion (AIC) variable selection. 1 = among top, 2 = very good, 3 = good. The lower table indicates the cut-off values used for classification as 1, 2 and 3 for BS_lasso, MeanAcc and Gini Impurity.

| Index | Lasso | BS_lasso | MeanAcc | Gini | AIC |
| --- | --- | --- | --- | --- | --- |
| CA125{1} | 1 | 2 | 2 | 3 |  |
| CA125{2} |  | 3 | 2 | 3 |  |
| CA125{3} | 1 | 1 | 1 | 1 | 1 |
| CA125{4} |  |  | 3 | 2 |  |
| CA125{5} | 1 | 1 | 1 | 3 | 1 |
| AGR2{3} |  |  |  |  | 1 |
| CHI3L1{3} |  | 3 | 3 | 3 | 1 |
| FSTL1{3} | 1 | 2 |  | 3 |  |
| FSTL1{5} |  |  |  |  | 1 |
| PEBP4{2} |  |  |  |  | 1 |
| PEBP4{4} |  |  |  |  | 1 |
| PEBP4{5} |  |  |  | 3 |  |
| HE4{1} |  | 3 | 3 | 3 |  |
| HE4{2} |  | 3 | 3 | 3 | 1 |
| HE4{3} |  | 2 | 2 | 2 |  |
| HE4{4} |  |  |  |  | 1 |
| HE4{5} |  |  |  |  | 1 |

|  | BS_lasso | MeanAcc | Gini |
| --- | --- | --- | --- |
| ‘Among top’ (1) | >0.85 | >14 | >7 |
| ‘Very good’ (2) | 0.3-0.85 | 9-14 | 3-7 |
| ‘Good’ (3) | 0.15-0.3 | 5-9 | 1-3 |

**Table S5**. McNemar’s test for comparison of top multimarker longitudinal models with CA125 cut-off model CA125{5}. *P* values are indicated (one-sided test) with shaded cells indicating *P* values <0.05.

| **Analysis** | **Model** | | | | |
| --- | --- | --- | --- | --- | --- |
|  | CA125{3}HE4{4}HE4{5} | CA125{3}AGR2{3}CHI3L1{3} | CA125{3}CHI3L1{3}HE4{5} | CA125{3}CHI3L1{3}HE4{1}} | CA125{3}CA125{4}PEBP4{5} |
| All cases; 1 year to diagnosis; >0.9 specificity | 0.035 | 0.035 | 0.055 | 0.055 | 0.016 |
| Type II cases; 1 year to diagnosis; >0.9 specificity | 0.016 | 0.016 | 0.016 | 0.016 | 0.031 |
| All cases; 1 year to diagnosis; >0.95 specificity | 0.09 | 0.055 | 0.055 | 0.145 | 0.063 |
| Type II cases; 1 year to diagnosis; >0.95 specificity | 0.031 | 0.016 | 0.016 | 0.063 | 0.125 |
| All cases; 1-2 years to diagnosis; >0.9 specificity | 0.344 | 0.302 | 1 | 0.754 | 0.688 |
| Type II cases; 1-2 years to diagnosis; >0.9 specificity | 0.5 | 0.274 | 0.5 | 0.5 | 0.5 |
| All cases; 1-2 years to diagnosis; >0.95 specificity | 0.109 | 0.5 | 0.5 | 0.5 | 0.313 |
| Type II cases; 1-2 years to diagnosis; >0.95 specificity | 0.5 | 0.5 | 0.5 | 0.25 | 0.5 |

**Supplementary Figure S1** Workflow for discovery and testing of candidate biomarkers and longitudinal models.

Candidate scoring

and selection

Sequential immunodepletion

(Proteome Purify 12 and ProteoPrep 20)

6-plex TMT

peptide level labelling

SAX and Basic RP-LC

(100 fractions)

Trypsin digest

**Discovery**

Pooled UKCTOCS samples in 6 groups: OC cases Type I/BL (n=19), OC cases Type II (n=30) and controls (n=31) in two pre-diagnosis time groups (‘late’ and ‘early’)

LC-MS/MS identification and relative quantification by TMT reporter ion intensity

Novel candidates CHI3L1, DNAH17, FSTL1, PEBP4 and LRG1

Reported markers CA125, HE4, SLPI, PAEP/glycodelin, AGR2

**Generation of Serial Data**

All individual samples from same 80 cases and controls (n=490)

**Model Generation and Testing**

- Indexing serial data (annual samples only at <5 yrs; n=338)
- Variable selection
- Model generation
- Leave-one-out cross-validation
- Goodness-of-fit testing
- Model performance (sensitivity at fixed specificity)
- Statistical comparison of models vs. CA125 alone
- Lead time estimates for test-positive cases

**Supplementary Materials and Methods**

*LC-MS/MS discovery analysis -* Equal volumes of relevant samples were pooled into six groups for MS-based discovery, comprising ‘late’ samples (taken <14 months to diagnosis) and ‘early’ samples (taken >35 months to diagnosis) for each ovarian cancer case and control group; Type I/BL early and late, Type II early and late, control early and late (see Table 1). For each pool, 15 µL of serum was depleted of abundant proteins using Proteome Purify 12 (R&D Systems, UK) and ProteoPrep 20 (Sigma-Aldrich, USA) sequentially, according to the manufactures’ instructions, yielding ~3% of the total starting material. Protein concentration was determined by Bradford assay and 100 µg was dried to near completion in a vacuum concentration. Samples were diluted in 100 µL of 100 mM triethyl ammonium bicarbonate (TEAB) at pH 8.5 and 0.1% SDS (w/v) and reduced with 1mM tris(2-carboxyethyl)phosphine (TCEP) for 1 hr at 55^o^C. Cysteines were blocked with 7.5 mM iodoacetamide for 1 hr at room temperature in the dark. Protein digestion was performed with sequencing grade modified porcine trypsin (Promega) at a ratio of 1:25 for 18 hrs at 37^o^C. TMT 6-plex differential labelling was performed by suspending 0.8 mg of TMT reagents (ThermoFisher Scientific) in 41 µL acetonitrile and combining with the 100 µg of peptide sample for 1 hr at room temperature. Labels were allotted as follows: 126 - Type II early; 127 - Type I early; 128 - control early; 129 - Type I late; 130 - control late; 131 - Type II late. Hydroxylamine was added to a concentration of 0.25% (v/v) for 30 min to block the reaction before samples were mixed (6-plexed). Detergent removal spin columns (Pierce) were used according to the manufacturer’s instructions to remove SDS, following which samples were dried before re-suspending in 2% (v/v) phosphoric acid and desalting with 1cc Oasis HLB cartridges (Waters). Samples were then dried and frozen at -20^o^C.

Peptides were fractionated firstly by strong anion exchange (SAX) (5 fractions) and secondly by reversed-phase liquid chromatography (RP-LC) at high pH (20 fractions) resulting in 100 fractions. For SAX, 600 µL Spin Cups (ThermoFisher Scientific) were filled with 300 µL of DEAE Ceramic HyperD F slurry (Pall Corp, USA) and centrifuged at 900 x g for 1 min before washing with 300 µL of 1M NaCl in 100 mM TEAB (pH 8.5) followed by 3 washes with 300 µL of 200 mM TEAB (pH 8.5) and equilibration with 100 mM TEAB (pH 8.5). The 600 µg of pooled labelled peptides were suspended in 300 µL of 100 mM TEAB and incubated with the equilibrated slurry for 50 min at room temperature with rotation. Unbound material was collected by centrifuging for 1 min and the beads washed with TEAB. The wash and flow through were combined (fraction 1). Peptides were then sequentially eluted with increasing concentrations of NaCl (0.05, 0.1, 0.2 and 1 M) in 100 mM TEAB by incubating with the buffer for 5 minutes with rotation and centrifuging for 1 min. Peptide fractions were acidified with phosphoric acid and desalted with 1cc Oasis HLB cartridges (Waters) by washing with 1 mL of 5% methanol and eluting with 100% methanol. Samples were dried and frozen at -20^o^C.

Further fractionation was achieved using high pH, RP-LC. SAX-fractions were suspended in 20 mM ammonium formate at pH 8.4 and loaded onto a PoroShell 300 Extend-C18 column (2.1 x 75 mm, 5 μm-bead size, 300 Å-pore size, Agilent) using an Agilent 1100 HPLC system. 20 fractions were collected by eluting with a gradient of acetonitrile (3-45%) over 35 minutes. Samples were dried to completion, re-solubilised in 200 µL 0.1% formic acid, dried down again and stored at -20^o^C.

Samples were re-suspended to a concentration of ~0.2 µg/µL in buffer A (0.1% formic acid) and LC-MS/MS performed, essentially as described (*40*). In brief, peptides were separated by C18 RP-LC on an Ultimate 3000 nano-liquid chromatography system (Dionex); repeat samples were injected onto an Acclaim PepMap 100 C18 pre-column (5 μm, 100 Å, 300 μm i.d x 5 mm) (ThermoFisher Scientific) and washed for 3 min with 10% buffer B (ACN + 0.1% (v/v) FA) at a flow rate of 25 μL/min and then peptides separated on an Acclaim PepMap 100 C18 Nano-LC column (3 μm-bead size, 100 Å-pore size, 75 μm i.d x 250 mm) (ThermoFisher Scientific) with a 90 min linear gradient of 10-50% buffer B at a flow rate of 300 nL/min. Repeat samples were electrosprayed into both LTQ-Orbitrap XL and LTQ-Orbitrap Velos instruments controlled using Xcaliber software (ThermoFisher Scientific). Parent MS scans were acquired in the orbitraps at a resolution of 60,000 at *m/z* 400, followed by top 3 CID/HCD (LTQ-Orbitrap XL) or top 10 CID/HCD (LTQ-Orbitrap Velos) using a normalised collision energy of 40% for HCD and a resolution of 7,500 for detection of product ions in the orbitrap. Dynamic exclusion was enabled with a list size of 500, excluding for 15 seconds. Atmospheric polymethylcyclosiloxane was used as a lock mass (455.12003 *m/z*) for in-run calibration.

Raw data files from both XL and Velos instruments were combined and analysed using Proteome Discoverer v1.4 software (ThermoFisher Scientific) with database searching against the UniProtKB/SwissProt database (2014_09; 546,439 sequence entries) using the Mascot search engine v2.4 (Matrix Science). For searching, taxonomy was human, MS tolerance was set to +/- 10 ppm, MS/MS tolerance was set to 0.5 Da and one missed cleavage was allowed. TMT 6-plex modification of peptides and carbamidomethylation of cysteines were set as fixed modifications. Protein N-terminal acetylation, methionine oxidation and N/Q deamidation were set as variable modifications. Search result filters were as follows: only peptides with a score of >20 and below the Mascot significance threshold filter of *P*<0.05 were included. Protein grouping was enabled such that when a set of peptides in one protein were equal to or completely contained within the set of peptides of another protein, the two proteins were put together into a protein group. The false discovery rate was calculated to be 2.3% based on searching a decoy database. Reporter ion-based quantification was carried using Proteome Discoverer Version v1.4 with the following 7 ratios calculated: Type I early vs. control early; Type II early vs. control early; Type I late vs control late; Type II late vs. control late; Type I late vs Type I early; Type II late vs Type II early and control late vs. control early.

A biomarker scoring system was applied to rank proteins to aid in candidate selection; protein groups were scored positively for fold-change between cases and controls, consistency of change between early and late time groups and MS data quality for identification and quantification. Negative scoring was applied if proteins had been targeted by immunodepletion or if proteins were known acute-phase reactants, apolipoproteins, clotting factors, complement proteins, immunoglobulins, or if the identification was from a single peptide. Proteins were ranked separately for Type I and Type II and for both types together. The full dataset is available as Supplementary Data File S1.

**Supplementary Data Files**

Supplementary Data File S1 - LC-MS based biomarker discovery and scoring matrix.xlsx
